# Expression of estrogen receptor beta in the breast carcinoma of BRCA1 mutation carriers

**DOI:** 10.1186/1471-2407-8-100

**Published:** 2008-04-13

**Authors:** Maria M Litwiniuk, Krzysztof Rożnowski, Violetta Filas, Dariusz D Godlewski, Małgorzata Stawicka, Remigiusz Kaleta, Jan Bręborowicz

**Affiliations:** 1 Clinic of Oncology, Poznan University of Medical Sciences, Poznan, Poland; 2 Prophylactics and Epidemiology Centre in Poznan, Poznan, Poland; 3 Private Oncological Practice, Gliwice, Poland

## Abstract

**Background:**

Breast cancers (BC) in women carrying mutations in BRCA1 gene are more frequently estrogen receptor negative than the nonhereditary BC. Nevertheless, tamoxifen has been found to have a protective effect in preventing contralateral tumors in BRCA1 mutation carriers. The identification of the second human estrogen receptor, ERβ, raised a question of its role in hereditary breast cancer. The aim of this study was to assess the frequency of ERα, ERβ, PgR (progesterone receptor) and HER-2 expression in breast cancer patients with mutated *BRCA1 *gene and in the control group.

**Methods:**

The study group consisted of 48 women with *BRCA1 *gene mutations confirmed by multiplex PCR assay. The patients were tested for three most common mutations of BRCA1 affecting the Polish population (5382insC, C61G, 4153delA). Immunostaining for ERα, ERβ and PgR (progesterone receptor) was performed using monoclonal antibodies against ERα, PgR (DakoCytomation), and polyclonal antibody against ERβ (Chemicon). The EnVision detection system was applied. The study population comprised a control group of 120 BC operated successively during the years 1998–99.

**Results:**

The results of our investigation showed that *BRCA1 *mutation carriers were more likely to have ERα-negative breast cancer than those in the control group. Only 14.5% of *BRCA1*-related cancers were ERα-positive compared with 57.5% in the control group (*P *< 0.0001). On the contrary, the expression of ERβ protein was observed in 42% of *BRCA1*-related tumors and in 55% of the control group. An interesting finding was that most hereditary cancers (75% of the whole group) were triple-negative: ERα(-)/PgR(-)/HER-2(-) but almost half of this group (44.4%) showed the expression of ERβ.

**Conclusion:**

In the case of *BRCA1*-associated tumors the expression of ERβ was significantly higher than the expression of ERα. This may explain the effectiveness of tamoxifen in preventing contralateral breast cancer development in *BRCA1 *mutation carriers.

## Background

In 1990 Hall et al. discovered that familial breast cancer is associated with a defect in one of the genes located in the 17q21 chromosome [1]. This finding began a new era of research into hereditary breast cancer and consequently led to the identification of the *BRCA1 *and *BRCA2 *suppressor genes in 1994 and 1995, respectively. Although the structures and localization of the *BRCA1 *and *BRCA2 *genes differ, their functions seem to be similar because their transcripts are involved in the same processes [2-6]. These genes are responsible for maintaining the proper course of the cell cycle, for the repair of DNA damage, and are also instrumental in the process of cell differentiation. *BRCA1 *is also partially responsible for the activity of estrogen receptors (ER) and, when mutated, can inhibit the functions of these receptors [7].

*BRCA1 *and *BRCA2 *gene mutation carriers are at risk of developing breast cancer earlier than other patients. Breast cancer associated with this mutation has characteristic histopathological features: (i) the expression of estrogen and progesterone receptors is less frequently demonstrable, (ii) the grade of histopathological malignancy is higher and (iii) accumulation of p53 protein is observed more often than in sporadic cases of this malignancy [8,9]. Although these factors are usually associated with a poorer prognosis, their role in *BRCA1 *and *BRCA2 *mutation carriers is still controversial [10-15].

The role of tamoxifen in preventing the development of contralateral breast cancer in *BRCA1 *mutation carriers is not fully understood since it significantly reduces that risk despite low expression of ER [16]. The mechanism responsible for that has not been yet explained and estrogen receptor β may play a role here.

Estrogen receptor β (ERβ) was discovered in 1996 and was given its name in order to differentiate it from the previously known type of estrogen receptor (now named estrogen receptor α – ERα) [17,18]. The two estrogen receptors belong to a family of ligand-regulated transcription factors. They are transcripts of different genes sharing some structural similarities. When co-expressed, ERα and ERβ may form homo- or heterodimers upon binding specific ligands. As dimers, ERs are able to start transcription activity in two ways: through direct binding to specific regions of DNA, or through protein-protein interaction with other transcription factors. In the case of co-expression of both ERs, their roles may overlap. In certain situations, however, ERβ opposes the activity of ERα via the inhibition of ERα-mediated gene expression. These differences are also observed in the response to tamoxifen. This selective estrogen receptor modulator may work as a pure ER antagonist for ERβ, while it may have a partially agonistic effect for ERα [19]. In spite of increasing knowledge regarding the structure and *in vitro *activity of ERα and ERβ, their clinical role is still controversial and unclear [20].

For a better understanding of the functions of ERβ we explored its expression in *BRCA1 *mutation carriers and looked for coexistence patterns with other hormonal receptors (ERα, PgR) and HER-2 receptor.

## Methods

The study group included 48 patients with mutations in the *BRCA1 *gene. The control group consisted of 120 subsequent breast cancer cases diagnosed over the period of 1998–1999. Patients from both groups underwent breast surgery from which specimens for histological and immunohistochemical testing were obtained. The study was approved by the local Bioethics Committee at the Medical University in Poznań.

Results of genetic tests were obtained from the Prophylactics and Epidemiology Center in Poznań. In the search for mutations in the *BRCA1 *gene, tests were performed on DNA isolated from peripheral blood lymphocytes using a commercially available kit. They were carried out for three most common mutations of *BRCA1 *gene affecting the Polish population (5382insC, C61G, 4153delA). In Poland those three mutations in BRCA1 account for 86% of all BRCA1 and BRCA2 mutations. In the search for 5382insC and 4153delA mutations, the ASA-PCR method was used. For the detection of C61G mutation, the RELP-PCR method was applied.

Histological and immunohistochemical tests were completed in the Department of Tumor Pathology of the Medical University in Poznań. From formalin fixed and paraffin embedded specimens, 4 μm sections were cut and mounted onto positively charged glass microscope slides (Superfrost Plus, Menzel-Glaser, Germany). The sections were deparaffinized, rehydrated and subjected to antigen retrieval in citrate buffer in a microwave oven. The slides were then incubated in 3% hydrogen peroxide for 10 minutes to block endogenous peroxidase activity.

The primary antibodies used for immunostaining were as follows:

1. for ERα – a monoclonal mouse antibody clone 1D5 (Dako, Glostrup, Denmark; code No M 7047) was used at 1:50 dilution and slides were incubated at room temperature for 1 h;

2. for ERβ – a polyclonal rabbit antibody in which immunogen corresponds to NH2-terminus of the human ERβ, and, according to the manufacturer, the sequence used is conserved in all known isoforms (Chemicon International, Temecula, CA; catalog No AB1410). This antibody was used at a dilution of 1:500 and overnight in an incubation chamber at 4°C;

3. for PgR – a monoclonal mouse antibody clone PgR636, (Dako, Glostrup, Denmark; code M 3569) was used at a dilution of 1:100 and slides were incubated at room temperature for 1 h. This antibody (according to the manufacturer) has been demonstrated by Western blot to react with both forms of the progesterone receptor: the PR-A and PR-B.

The antibody reactions were revealed using Dako EnVision™+System-HRP. Subsequently the slides were incubated in DAB chromogen for 5 minutes at room temperature. The sections were counterstained with hematoxylin, and coverslipped.

Immunostained slides were evaluated by two independent observers in a "blinded" fashion, using light microscopy. Tumors were considered to be expressing receptors if a positive reaction (regardless of its intensity) was identified in at least 10% of the cancer cells' nuclei.

HER-2 protein was identified by means of the HecepTest™ kit (Dako). Tumors were classified as 0, 1+, 2+ or 3+ on the basis of the spread and intensity of membranous staining in invasive portions of the cancer. Tumors showing staining reactions in class 3+ were considered to be over-expressing HER-2. In the case of the 2+ results obtained, suggestive of over-expression of the HER-2 protein, amplification of the *c-erbB2 *gene was tested by the application of fluorescent in-situ hybridization (FISH). Cases with an immunohistochemistry result of 2+ but without amplification of the gene, as tested by FISH, were not considered to be over-expressing HER2.

To assess dependences Fisher' Exact Test (for 2 × 2 contingency tables) and the Fisher-Freeman-Halton (for larger contingency tables) test were applied. Significance was accepted at *P *< 0.05.

## Results

The clinicopathological characteristics of breast cancer patients with mutated *BRCA1 *gene and those of the control group are shown in Table 1. *BRCA1 *gene mutations in the study group were identified as follows: 5382insC (77.5%), C61G (20.4%) and 4153delA (2%).

**Table 1:** Clinicopathological characteristics of patient cohorts

Parameter	BRCA1-associated breast cancers n = 48	Control group n = 120	*P*-value
Age			
median (range)	45 (29 – 68)	57 (38 – 85)	<0.0001
Age > 50 years	16 (33%)	82 (68%)	
			
Histological type			
invasive ductal	42 (87%)	103 (85.8%)	0.7993
invasive lobular	1 (2%)	10 (8.3%)	0.14
medullary like	5 (10%)	0	< 0.001
other	0	9 (7.5%)	< 0.001
			
Tumor grade			
I	1 (2.0%)	36 (29%)	<0.001
II	11 (22.9%)	53 (44.1%)	0.0122
III	27 (56.2%)	26 (21.7%)	<0.0001
unknown	9 (18.7%)	5 (4.2%)	0.0017
			
Tumor size			
median	2.2	3.0	0.14
≤ 2 cm	28 (58.3%)	53 (44.1%)	0.102
> 2 cm ≤ 5 cm	16 (33.3%)	50 (41.6%)	0.282
> 5 cm	3 (6.2%)	17 (14.1%)	0.147
unknown	1 (2.0%)	0	
			
Lymph node			
negative	34 (70.8%)	52 (43.3%)	0.001
positive ≤ 3	8 (16.6%)	38 (31.6%)	0.015
positive >3	4 (8.3%)	22 (18.0%)	0.104
unknown	2 (4.1%)	8 (6.6%)	
			
Bilateral breast cancer	13 (27.1%)	2 (1.7%)	<0.0001
			
Mutation:			
5382insC	37 (77.5%)		
C61G	10 (20.4%)		
4153delA	1 (2%)		

The average age of patients with mutations in the *BRCA1*gene was 45 years, and 57 years in the control group.

Invasive ductal carcinoma (no special type) was found to occur at the same frequency in both the *BRCA1*-positive group as well as the control group (87% and 85.8%, respectively). Other tumor types were much less frequent, although it is noteworthy that all 5 patients with "medullary like" carcinoma came from the *BRCA1 *mutation carriers group (10%).

The assessment of histological malignancy was performed according to the Bloom-Richardson classification and showed that patients carrying *BRCA1 *mutations developed grade 3 tumors more often and had less frequent metastases to axillary lymph nodes.

In the *BRCA1*-positive group, the rate of patients who developed bilateral breast cancer accounted for 27.1% (13 cases). In the control group, bilateral breast cancer was found in 2 patients only (1.7%).

**Figure 1:**
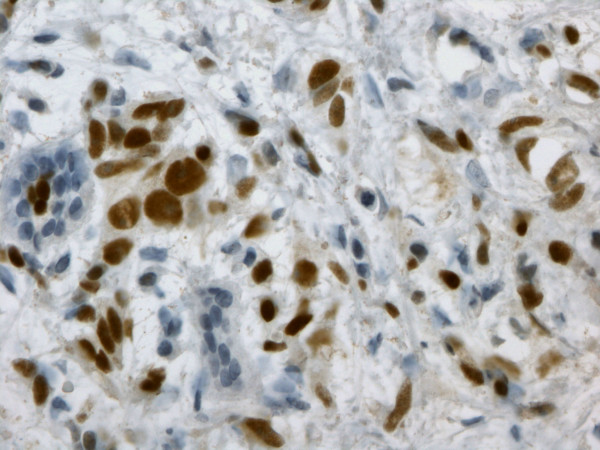
ERα immunostaining on paraffin-embedded invasive breast cancer using 1D5 Dako antibody (40× magnification).

The presence of estrogen receptor α was detected in the tumors of 7 (14.5%) patients with *BRCA1*gene mutations and in 69 patients (57.5%) in the control group (Fig. 1).

**Figure 2:**
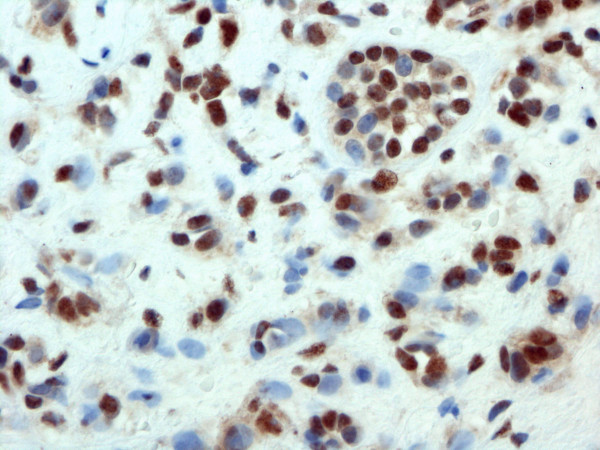
ERβ immunostaining on paraffin-embedded invasive breast cancer using antibody from Chemicon (40× magnification).

For progesterone receptors, the figures were 6 (12.5%) and 77 (64%), respectively. The expression of estrogen receptor β was detected in the tumors of 20 (42%) of the *BRCA1*-mutation patients and in 66 (55%) of the subjects in the control group (Table 2) (Fig. 2).

**Table 2:** Receptor status in BRCA1-associated breast cancer and in cancer of the control group

Expression of steroid receptors	BRCA1-associated breast cancers n = 48		Control group n = 120		*P*-value
	n	(%)	n	(%)	
ERα(+)	7	14.5	69	57.5	<0.0001
ERβ(+)	20	42	66	55	0.129
PgR(+)	6	12.5	77	64	<0.0001
HER2(+)	3	6.2	20	16.7	0.064

In the group with *BRCA1 *mutations, the most frequently found phenotype was ERα(-)/ERβ(-) observed in 52% of patients, followed by ERα(-)/ERβ(+) noticed in 33.3% cases. In the control group only 14.2% of the patients presented phenotype ERα(-)/ERβ(+). The most common phenotype observed in that group was ERα(+)/ERβ(+) – 40.8% (Table 3).

**Table 3:** The distribution of ERα and ERβ in BRCA1-associated breast cancers and in cancers of the control group

Receptors' status	BRCA1-associated breast cancers n = 48		Control group n = 120		***P***
	N	(%)	N	(%)	
ERα(+)/ERβ(+)	4	8.3	49	40.8	<0.0001
ERα(+)/ERβ(-)	3	6.2	20	16.7	0.086
ERα(-)/ERβ(+)	16	33.3	17	14.2	0.009
ERα(-)/ERβ(-)	25	52	34	28.3	0.004

The over-expression of the HER-2 receptor in patients with mutated *BRCA1 *genes was observed only in 3 cases (6.2%). In the control group over-expression of HER-2 was found in 20 cases (16.7%).

An interesting finding of this study is that most hereditary cancers have been found to be triple-negative: ERα(-)/PgR(-)/HER-2(-). Such a phenotype was found in 36 cases, which accounts for 75% of the whole group of patients with a mutated *BRCA1 *gene. 16 patients in that group expressed ERβ and presented the following phenotype: ERα(-)/PgR(-)/HER2(-)/ERβ(+). In the control group, the triple-negative phenotype was found in 24 patients (20%) of whom 5 had tumors expressing ERβ.

## Discussion

In our study, patients with *BRCA1 *gene mutations were tested for the expression of steroid receptors (ERβ, ERα and PgR) and the HER-2 receptor in breast cancer tissue. In this group, ERβ was more common (42%), while ERα and PgR occurred less frequently (14.5% and 12.5% respectively). In the control group, the expression of ERα and PgR was 57.5 and 64%, respectively. In the case of ERα and PgR, the differences between the two groups were found to be statistically significant (p = 0.001). Those values are similar to those described by other authors [8,12,21,22]. In the case of ERβ, however, the differences of expression between those two groups were small.

Most of our control group patients were tested for the presence of BRCA1 mutations. Although those tests were not performed on all the controls, this cannot influence the overall findings of the study since in Poland BRCA1 mutations (unselected for age) occur only in about 3% of all breast cancer patients [23].

It has been pointed out in many studies that *BRCA1 *positive breast cancers rarely express ERα [8-12,20,21]. Further confirmation of that observation comes from recent studies made with the use of the cDNA microarray technique, showing that breast cancers may vary considerably in their molecular profile [24,25]. The largest group consists of tumors with a cellular profile matching that of the inner layers of the mammary glands, such as luminal cells (luminal types) and another group consists of tumors with basal cell profiles (basal types). These two groups differ in their expression of ERα. The luminal types express ERα, while the basal types usually do not. cDNA studies of breast cancers with underlying *BRCA1 *gene mutations show that the most common cancer type in this group is basal [26]. Moreover, basal type tumors show expression of receptors for epidermal growth factor (EGFR) more often. Generally, about half of the patients with *BRCA1 *gene mutations present the phenotype ER(-)/HER2(-)/EGFR(+) of the basal type of tumor, which is usually associated with poor prognosis [26-28]. On the other hand, there are data that suggest that the prognosis for *BRCA1*-positive patients is no worse than that for patients without mutations [10-14]. This discrepancy may suggest the influence of some incompletely explored prognostic and predictive factor(s).

Recent analyses suggest that ERβ may be an independent prognostic and predictive factor in the course of breast cancer [29-33]. However, its role has not yet been fully discovered. It is noteworthy that in our clinical material (in contrast to ERα) the expression of the ERβ in tumor tissues of patients with *BRCA1 *gene mutations is almost as frequent as in the non-hereditary breast cancer patients. It would be interesting to test whether frequent representation of ERβ in *BRCA*-positive tumors is actually responsible for the earlier reported activity of tamoxifen in the reduction of contralateral breast cancer risk.

Fan et al. showed that there is a link between estrogen receptors and the BRCA1 protein. In an *in vitro *study, BRCA1 protein proved to be one of the transcription regulators for active ERα. The transcription co-activator p300 plays an important role in this process, and its presence correlates with the ability of the BRCA1 protein to suppress the activation of ERα transcription [7].

Our study group displayed certain characteristics of hereditary breast cancer. One of these features was the young age of the patients. In this group, the average age of breast cancer diagnosis was 45 years, 12 years earlier than in the control group. The same average age at which the diagnosis was established, was reported for Spanish families and a similar age (43 years) in Markus's study of the population of Ashkenazi Jews [10]. Age is a well known factor influencing the expression of ERα. The fact that there was a 12-year difference in the average age between the *BRCA1 *gene mutations group and the control group must have some influence on the reported ERα expression in our study. Regarding ERβ expression, however, we believe that difference bears no material significance on the result. Our opinion is based on the fact that numerous studies have indicated that the expression of ERβ is not age-dependent.

In both groups, the most frequent histological diagnosis was that of ductal carcinoma (87% and 85.8%, respectively). Another common histological type of breast cancer found in the mutation carriers group was "medullary like" carcinoma (10%). In the control group, however, not a single case of that type of cancer was identified. A higher rate of "medullary like" carcinoma is typical of *BRCA1 *mutation carriers and this diagnosis is associated with a relatively good prognosis [34,35]. Consequently, it was believed that this diagnosis positively affected the prognosis for *BRCA1*-positive patients. Unfortunately, a recent study failed to confirm that finding [36].

Patients with hereditary disease have been found to develop bilateral breast cancer more often. Our study has confirmed this observation as it has been found that bilateral breast cancer affected 13 patients of the study group (27.1%).

The above characteristics of hereditary breast cancer have been frequently described in the literature and are consistent with the previously published data. Little, however, has been said regarding the expression of ERβ. This appears to have been mentioned only once in a letter to the Editor of the Journal of Clinical Oncology which reported on estrogen receptor-beta expression in hereditary breast cancer where positive staining for ERβ was detectable in 94% (15 of 16) of *BRCA1 *associated breast cancers [37].

In our study, ERβ expression was present in 42% of *BRCA1 *mutation carriers. This seems to be important as it may explain the protective effect of tamoxifen in the prevention of contralateral tumor development in *BRCA1 *mutation carriers.

Out of the hereditary cancer patients in our study group, 75% were found to be triple-negative, lacking ERα, PgR and HER-2 over-expression. Unfortunately, at the current state of the art, neither hormonal therapy, nor immunotherapy (trastuzumab) in an adjuvant setting, can be offered to those patients.

Gruvberger-Saal et al [38] reported that the expression of ERβ is an independent marker for favourable prognosis after adjuvant tamoxifen treatment in ERα negative breast cancer patients. Bearing in mind that as many as 44% of the triple-negative patients in our study expressed ERβ, we propose that hormone therapy might be used in the treatment of *BRCA1 *mutation carriers expressing the ERα(-)/PgR(-/HER2(-)/ERβ(+) phenotype.

The results obtained in our study show that the expression of ERβ in *BRCA1 *gene mutation carriers is statistically higher than the expression of ERα. This may be why tamoxifen has proven to be effective in preventing the development of contralateral breast cancer in *BRCA1 *mutation carriers. Consequently, testing patients carrying *BRCA1 *mutations for the presence of ERβ might help to identify those who could benefit from endocrine therapy.

## Conclusion

In the case of *BRCA1*-associated tumors the expression of ERβ was significantly higher than the expression of ERα. This may explain the effectiveness of tamoxifen in preventing contralateral breast cancer development in *BRCA1 *mutation carriers.

## Abbreviations

ER – estrogen receptor

ERα – estrogen receptor alpha

ERβ – estrogen receptor beta

PgR – progesterone receptor

*BRCA1 *– breast cancer associated gene

*BRCA2 *– breast cancer associated gene

PCR – polymerase chain reaction

HER-2 – human epidermal growth factor receptor-2

SERM – selective estrogen receptor modulator

IHC – immunohistochemistry

ASA-PCR – amplicon sequence analysis – polymerase chain reaction

RFLP-PCR – restriction fragment length polymorphism – polymerase chain reaction

FISH – fluorescent hybridization in situ

EGFR – epidermal growth factor receptor

cDNA – complementary DNA

## Competing interests

The author(s) declare that they have no competing interests.

## Authors' contributions

ML conceived, designed and performed the study, coordinated the work and drafted the manuscript, KR participated in the draft of the manuscript, VF performed IHC tests, DG and MS carried out genetic tests, RK reviewed the manuscript and offered some comments, JB interpreted histopathological and IHC test results. All authors have read and approved the final manuscript.

## Pre-publication history

The pre-publication history for this paper can be accessed here:


